# Associations Between Delousing Practices and Pasteurellosis in Farmed Atlantic Salmon

**DOI:** 10.1111/jfd.14085

**Published:** 2025-01-27

**Authors:** Leif Christian Stige, Duncan J. Colquhoun, Victor H. S. Oliveira

**Affiliations:** ^1^ Norwegian Veterinary Institute Ås Norway

**Keywords:** Atlantic salmon ( *Salmo salar* ), *Lepeophtheirus salmonis*, *Pasteurella*, sea louse treatment

## Abstract

Infections with bacteria of the genus *Pasteurella* have increased in occurrence in Atlantic salmon ( *Salmo salar* ) farms in Norway since 2018. This increase coincides with increased use of non‐medicinal treatments against the parasitic salmon louse, *Lepeophtheirus salmonis* , in the farms. Here, we analysed the statistical association between the use of non‐medicinal delousing methods and pasteurellosis in salmon farming in western Norway, from 2018 to 2023. The analysed data covered 1161 production cycles from 356 farming localities, in which *Pasteurella* was detected in 166 production cycles from 115 localities. Results showed that in months with one or more thermal delousing using heated water to remove the lice, the odds for detection of *Pasteurella* were 2.5 times as high as in months with no delousing (95% credible interval, c.i.: 1.8, 3.6). In months with one or more mechanical delousing involving brushing or flushing, the odds were 1.8 times as high as in months with no delousing (c.i.: 1.04, 2.9). Delousing with freshwater was not associated with increased odds for *Pasteurella*. The odds for *Pasteurella* increased with increasing salmon weight and following a *Pasteurella* diagnosis in the previous production cycle. These results indicate that the use of thermal and mechanical delousing may play a role in maintaining the current pasteurellosis situation in western Norway, and that freshwater delousing may be a favourable alternative in this respect.

## 1 Introduction

Infections in Atlantic salmon sea‐farmed in Norway, associated with members of the genus *Pasteurella* (Birkbeck et al. 2002), have been recognised for many years. Such infections are almost exclusively caused by the as‐yet officially un‐named ‘*Pasteurella atlantica*’ (Nilsen, Gulla, and Colquhoun 2024), although *P. skyensis* has also been reported (Strøm and Nilsen 2021). Since the first reported diagnosis in 1989 (Valheim et al. 2000), *Pasteurella* infections have been extremely limited in terms of both frequency and impact (Gulla et al. 2023). Since 2018, however, the situation has changed dramatically for the worse, with around 50 outbreaks reported annually between 2019 and 2022 (Nilsen, Gulla, and Colquhoun 2024). The reasons behind this apparent endemic establishment of the disease within a geographically limited area of western Norway are not clear. Each chronologically disparate outbreak of pasteurellosis in Norwegian farmed salmon has been associated with a unique genotype of ‘ *P. atlantica* subsp. *salmonicida*’ (Gulla et al. 2023). Results from controlled infection trials (D.J. Colquhoun, unpublished) do not suggest that the present genotype circulating in the current zoonotic is particularly infectious or virulent.

The salmon louse, *Lepeophtheirus salmonis* , is arguably the most important pathogen in Norwegian aquaculture. Widespread resistance to chemotherapeutic treatments has resulted in a shift towards non‐medicinal delousing methods (Myhre Jensen et al. 2020; Sommerset et al. 2024). These non‐medicinal methods include ‘thermal delousing’ that is, exposing the fish to heated water for around 20–30 s, ‘mechanical delousing’ that is, removal of lice by brushing and/or water jets and ‘freshwater delousing’ that is, exposure of the fish to water of low salinity (Overton et al. 2019). All these methods include crowding and handling of the fish and have been associated with increased acute and delayed mortality (Oliveira et al. 2021; Overton et al. 2019; Sviland Walde et al. 2021; Tvete, Aldrin, and Jensen 2023), reduced growth (Sviland Walde et al. 2022) and reduced welfare (Thompson et al. 2024; Østevik et al. 2022). Anamneses received by the diagnostic service of the Norwegian Veterinary Institute (NVI) commonly mention that clinical disease associated with *Pasteurella* infection follows non‐medicinal delousing.

The objective of this study was, therefore, to investigate the association between the use of non‐medicinal delousing methods in salmon farming and pasteurellosis. We hypothesised the risk of pasteurellosis to be higher in farms and periods of the production cycle when thermal, mechanical or freshwater delousing was performed, while accounting for the influence of potential confounding factors that might cause spatiotemporal co‐variation between delousing and pasteurellosis.

## 2 Materials and Methods

### 2.1 Data Sources

Pasteurellosis is a non‐notifiable disease in Norway, meaning its diagnosis is not mandatory reported to the authorities. However, aquaculture companies routinely submit biological samples to the NVI or private laboratories for diagnostic investigation. The NVI serves as the national reference laboratory for fish diseases. For this study, diagnostic data for pasteurellosis were sourced from the NVI between 2018 and 2020, which was probably the laboratory receiving most of the related sample submissions during that time. From 2021 to 2023, the data collection expanded to include four private laboratories due to an increase in sample submissions to these laboratories. Diagnoses were confirmed using culture, PCR or immunohistochemistry‐based methodologies either alone or in combination. Diagnostic samples were derived from individual fish, with the sample numbers likely varying across farms. We considered all cases with a laboratory‐confirmed *Pasteurella* infection as positive, regardless of the presence of clinical signs suggestive of pasteurellosis at the farm. The data from the multiple sources was standardised into a unified structure and combined, providing a comprehensive retrospective overview of *Pasteurella* cases in Norway from 2018 to 2023.

Delousing data was obtained from the Norwegian Food Safety Authority (NFSA). Salmonid fish farmers in Norway are required to report lice counts weekly to the NFSA and at the same time report whether medicinal or non‐medicinal delousing has been utilised that week (Ministry of Trade, Industry and Fisheries 2012). Until 2023, the method used for non‐medicinal delousing was described in a free‐text field, while during 2023, a new reporting form was introduced, in which the delousing method may be categorised as thermal, mechanical or freshwater. To categorise free‐text‐based entries, we used an R‐script to search for text segments that uniquely identified the method used as thermal, mechanical or freshwater (Qviller, Stige, and Helgesen 2024). These text identifiers are listed in Table S1.

Salmon production data was obtained from the Norwegian Directorate of Fisheries. This dataset describes the number and mean weight (kg) of salmon held in each farm at the end of each month. As farms holding salmon grown for purposes other than food‐fish production may not be required to report on salmon lice numbers or delousing methodology, we here limited our analyses to data from farms that reported both salmon lice number and delousing method in the study period from 2018 to 2023.

Handling of data was performed using R software version 4.0.3 (R Core Team 2020) and the Tidyverse package collection version 2.0.0 (Wickham et al. 2019).

### 2.2 Statistical Analysis

**FIGURE 1:**
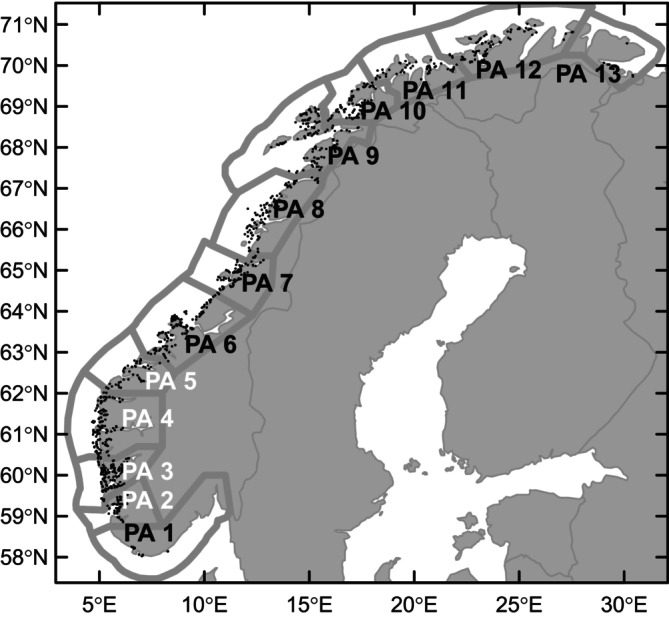
Study area. The Norwegian coast is divided into 13 aquaculture production areas (PA), of which *Pasteurella* has been identified in marine farmed Atlantic salmon from PA 2 to 5. Active marine salmon farms are shown by black dots.

To identify possible associations between the use of non‐medicinal salmon‐louse treatments and pasteurellosis, we analysed monthly data reported from marine salmon farms in production areas 2 to 5 (Figure 1) from the years 2018–2023. The analysed data covered 1161 production cycles from 356 farming localities, in which *Pasteurella* was detected in 166 production cycles from 115 localities. For the years 2018–2020 and 2023, the dataset covered all active marine salmon farms in the area, whereas for 2021 and 2022, the dataset excluded one company that did not grant us permission to use their data for these years (representing 5% of the localities).

The binary response variable in the analysis was detection or not of *Pasteurella* in a given month and farm. For production cycles with detection of *Pasteurella*, data was included only up to and including the month of initial detection. A total of 13,912 farm‐months were analysed.

The probability of detection of *Pasteurella* was analysed by mixed‐effects logistic regression, which modelled the odds for the probability of detecting *Pasteurella* in a given farm *f* and month *t*:

(1) $${\text{ODDS}}_{f,t}=\frac{\text{Prob}\left({\text{Detection}}_{f,t}\right)}{1-\text{Prob}\left({\text{Detection}}_{f,t}\right)}$$

The natural logarithm of the odds was considered an additive function of the predictor variables, implying multiplicative effects on the odds scale. The predictors included a louse treatment variable, variables for potentially confounding factors, and random effects for farm locality and production cycle. The equation for the main model was:

(2) $$\begin{array}{c} \ln\left({\text{ODDS}}_{f,t}\right)=f\left({\text{Year}}_{t}\right)+g\left({\mathrm{PA}}_{f}\right)+h\left({\text{Weight}}_{f,t}\right)+i\left({\text{Prev}}_{f,t}\right)\\ \;+j\left({\text{Treat}}_{f,t}\right)+\varphi_{f}+\delta_{f,t} \end{array}$$

Here, *f*(*Year*) is a categorical function of year (2018, …, 2023), *g*(*PA*) is a categorical function of production area (2, 3, 4 and 5), *h*(*Weight*) is a categorical function of mean salmon weight (0–1, 1–2, 2–3, 3–4 and > 4 kg), *i*(*Prev*) is a categorical function of whether or not *Pasteurella* was detected in the previous production cycle on the farm, *j*(*Treat*) is a function of the use of different types of non‐medicinal salmon louse treatment the previous month (as detailed in the next paragraph), *φ* is a normally distributed farm effect, and *δ* is a normally distributed production cycle effect (nested in *φ*). Categorical functions were chosen for all covariates as we did not succeed in making models with continuous functions converge.

The treatment function was coded as three categorical functions, one for each treatment type (i.e., thermal, mechanical and freshwater), meaning that if more than one treatment type was used in the same month, the effects were additive. The data was analysed at a monthly scale, but the data also contained information about the dates of *Pasteurella* diagnosis and louse treatments. For *Pasteurella*‐positive months, the *Treat* variable(s) indicated whether or not each of the treatment types was applied at least once during the 31 days preceding and including the diagnosis date. For *Pasteurella*‐negative months, the *Treat* variable was scored based on the treatment data for the given calendar month (with no time lag).

To assess the robustness of the main results to the choice of covariates, we also considered an alternative model formulation that, in addition to the terms in Equation (2) included a categorical function of calendar month (January, February, …, December) and an alternative model formulation that replaced the function of salmon weight with a categorical function of time in the production cycle (months 1–3, 4–6, 7–9, 10–12, 13–15 and 16+). We also built a model that tested if the probability of detecting *Pasteurella* varied between production cycles with different fallow periods following a *Pasteurella*‐positive production cycle. In this model, *i*(*Prev*) was a categorical function that estimated different effects for different fallow periods: no *Pasteurella* in the previous production cycle; or *Pasteurella* and 4–8, 9–12 or 13+ months of fallowing.

The parameters in the model were estimated in a Bayesian framework using the software Stan and the R interface ‘brms’ version 2.19.0 (Bürkner 2017; Stan Development Team 2023), and R version 4.0.3. Priors were default non‐ or very weakly informative. The joint distribution of the parameters were estimated in four parallel Hamiltonian Monte Carlo chains (Stan Development Team 2023) with 4000 iterations each, of which the first half was discarded as ‘burn‐in’. The statistical significance of model parameters was assessed from 95% credible intervals calculated from this distribution. To avoid so‐called divergent transitions, an indication of estimation problems (Stan Development Team 2023), the adapt_delta parameter was set to 0.99. Model convergence was confirmed by visual inspection of the chains and by summary statistics such as Gelman and Rubin $\hat{R}$ convergence diagnostics (Gelman and Rubin 1992).

## 3 Results

### 3.1 Overall Patterns

**FIGURE 2:**
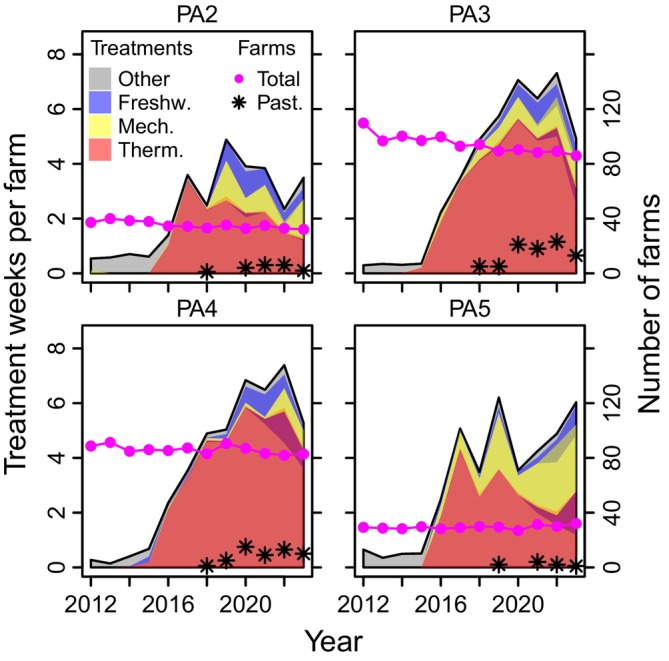
Trends in non‐medicinal delousing per farm, number of active farms and number of farm production cycles with detection of *Pasteurella* (Past.) per production area (PA). The number of delousing treatments refers to weeks per farm with a reported non‐medicinal treatment against salmon lice, categorised into thermal (‘Therm’), mechanical (‘Mech’), freshwater (‘Freshw.’) and other. Overlapping colours represent farm‐weeks with more than one treatment type reported. Only production areas with *Pasteurella* cases are shown.

From 2018 to 2023, *Pasteurella* was diagnosed in marine farmed salmon in production areas 2–5, with 83% of cases in production areas 3 and 4 (Figure 2). The number of cases increased from 2018 to 2020, was relatively stable until 2022, before decreasing in 2023. In the same area and period, non‐medicinal salmon louse treatments have been frequently used, particularly thermal, but also treatments utilising flushing and brushing (‘mechanical’) or freshwater bathing (Figure 2).

### 3.2 Statistical Analysis

**FIGURE 3:**
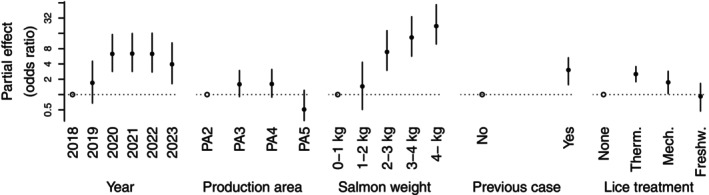
Estimated covariate effects on the monthly probability of *Pasteurella* in a salmon farm. The covariates were year, production area, mean salmon weight, whether or not *Pasteurella* had been diagnosed in the preceding production cycle at the farm, and whether or not a thermal, mechanical or freshwater salmon louse treatment had been used during the last month. Effects are shown as odds ratios, which are the ratios for the odds of *Pasteurella* for a given covariate state compared to a reference state (indicated by a circle for each covariate). An odds ratio of 1 indicates no difference in odds of *Pasteurella*. Bands show 95% credible intervals.

The statistical analysis quantified the association between *Pasteurella* cases and the use of non‐medicinal salmon louse treatments while accounting for the effects of year, production area, salmon weight and the detection of *Pasteurella* in the previous production cycle. Results showed significant associations between the use of thermal or mechanical delousing and *Pasteurella* cases, but not between freshwater delousing and *Pasteurella* (Figure 3). In months with one or more thermal delousing, the odds for *Pasteurella* were 2.5 times as high as in months with no delousing (95% credible interval, c.i.: 1.8, 3.6). In months with one or more mechanical delousing, the odds were 1.8 times as high as in months with no delousing (c.i.: 1.04, 2.9). For example, a farm in production area 3 in 2023, with a mean salmon weight above 4 kg and no *Pasteurella* diagnosis in the previous production cycle, had 1.7% probability of *Pasteurella* in a month without non‐medicinal louse treatments. This probability increased to 4.4% following a thermal treatment and 2.9% after a mechanical treatment in the preceding month. If the farm had *Pasteurella* diagnosed in the previous production cycle, these probabilities rose to 5.2%, 13.1% and 9.0%, respectively.

The analysis further showed increasing odds of *Pasteurella* between 2018 and 2020, stability between 2020 and 2022, with indication of decreased odds between 2022 and 2023 (Figure 3). The odds were highest in production areas 3 and 4, lower in production area 2 and lowest in production area 5. The odds increased with the mean weight of the salmon, with 1.5 times higher odds at 1–2 kg compared to < 1 kg, 6.9 times higher at 2–3 kg, 13 times higher at 3–4 kg and 22 times higher at > 4 kg (compared to at < 1 kg). The odds for *Pasteurella* were 3 times higher in farms with a positive diagnosis during the previous production cycle compared to farms not found positive in the previous cycle. Summary statistics of the main model, including posterior means and standard errors of all parameters, are included in Table S2.

The treatment effects estimated by a model accounting for seasonality by including calendar month as a covariate were similar to those estimated by the main model (Figure S1). The estimated treatment effects were also similar in models including either production month (alternative model) or mean salmon weight (main model) as covariates, except that the effect of mechanical treatments changed from marginally significant in the main model to marginally non‐significant in the alternative model (Figure S2). With this model formulation, the odds for *Pasteurella* were 2.4 times as high following a thermal treatment compared to no treatment (c.i.: 1.6, 3.4) and 1.7 times as high following a mechanical treatment compared to no treatment (c.i.: 0.93, 2.8). A model that included fallowing after a previous diagnosis as covariate revealed no difference in risk between 4–8, 9–12 and 13+ months of fallowing (Figure S3). The estimated treatment effects in the model including fallow period as covariate were similar to the main model.

## 4 Discussion

Since 2018, pasteurellosis has become a disease of serious concern to the Norwegian salmon farming industry. The reasons behind the re‐emergence of this disease and its transition from an occasional issue in previous years to an apparently geographically limited but endemic problem, remain unclear. This development does, however, chronologically overlap with the introduction and widespread adoption of non‐medicinal delousing methodologies within the Norwegian aquaculture industry. Due to widespread resistance in salmon lice to existing medicinal treatments (Aaen et al. 2015) and a lack of alternative treatments, these non‐medicinal methodologies now dominate (Myhre Jensen et al. 2020; Sommerset et al. 2024), despite their acknowledged negative effects on fish health and welfare (Moltumyr et al. 2022; Thompson et al. 2024; Østevik et al. 2022).

Here we analysed delousing practices and diagnostic data from active marine salmon farms in PAs 2–5 (the affected area) over a 5‐year period. We hypothesised the risk of pasteurellosis to be higher in farms and periods of the production cycle when thermal, mechanical or freshwater delousing were performed. Our hypothesis was partially supported, as the analysis revealed statistically significant associations between delousing using thermal or mechanical methods and subsequent identification of *Pasteurella* infection in treated populations of salmon, while the same significant association could not be identified for delousing strategies utilising freshwater bathing.

Non‐medicinal delousing practices and thermal delousing in particular, have been demonstrated to result in elevated mortality at least 1–2 weeks post treatment (Sviland Walde et al. 2021; Tvete, Aldrin, and Jensen 2023) through direct physical injury caused by collision with treatment furniture etc. Such injuries in surviving fish undoubtedly offer opportunities for colonisation of open lesions by various infectious agents. Previous research has demonstrated increased accumulation of both *Yersinia ruckeri* and ‘*Pasteurella atlantica*’ in treatment baths when sub‐clinically infected fish are subjected to thermal delousing (Riborg et al. 2022; Strand et al. 2023). Additionally, eDNA‐based detection of ‘ *P. atlantica* ’ associated with mechanical delousing has been shown to have predictive value related to forthcoming pasteurellosis outbreaks (Strand et al. 2023). These findings support a causal basis for the positive association between *Pasteurella* infections and thermal and mechanical (brushing or flushing) treatment methods. In contrast, despite the fact that crowding and pumping of fish are common across non‐medicinal delousing methods, there was a lack of a significant association between freshwater delousing and later identification of *Pasteurella* within treated stocks. There may be several plausible explanations for this. Freshwater‐based delousing, while not stress‐free, is probably less stressful than alternative non‐medicinal delousing methodologies (Thompson et al. 2024). This could be related to the fact that freshwater treatments can be performed within sea cages, thus reducing the need for fish pumping and handling. Such differentiation for the procedures of freshwater treatments has not been evaluated in this study. We have found ‘ *P. atlantica* ’ to be relatively poorly transmissible from salmon to salmon under controlled and comparatively stress free laboratory conditions (D. J. Colquhoun, unpublished). As all infectious diseases are dependent on the balance between the pathogenic agent, the susceptibility of the host to the pathogenic agent and the environment in which the pathogenic agent and the host find themselves, it may be that overall stress levels (or treatment associated lesions) do not surpass a necessary threshold during freshwater‐based delousing. Perhaps more significantly, ‘ *P. atlantica* ’ has been found to become inactivated in freshwater within 60 min or less of exposure (H. Nilsen, pers. comm.). This suggests that this bacterium, even if shed from subclinically infected fish, may not survive long enough in freshwater to infect näive fish.

The results also showed that the odds of *Pasteurella* infection increased with fish size, or alternatively with time in the production cycle (as also noted by Sandlund et al. 2021). This finding suggests that the risk of pasteurellosis is higher for older and larger fish, either because the susceptibility to the disease increases as fish age and grow, or because the establishment of an infection requires a prolonged exposure to risk factors in the farm environment and/or prolonged exposure to the infective agent.

The odds for *Pasteurella* were higher if *Pasteurella* had been diagnosed in the previous production cycle at the farm location. One interpretation of this finding is that the bacterium had survived in the farm environment from one production cycle to the next. It is unclear what could have been the reservoir for *Pasteurella* in the fallow period, and we note that longer fallowing did not reduce the odds significantly. Alternatively, the reservoir may have been fish farms in epidemiological contact with the given farm, through e.g., using the same well boat for delousing. Finally, the association may reflect some unknown factor that caused increased risk in both the previous and the current production cycle, so that a previous diagnosis acted as a proxy for this factor in the analysis. No matter which of these interpretations is correct, we note that a diagnosis of *Pasteurella* in a farm probably predicts increased risk in a subsequent production cycle.

An alternative model showed a peak of *Pasteurella* cases in January compared to all other months of the year. We find it unlikely that this association reflects a true seasonal trend, as there are no indications of increased odds for *Pasteurella* in the other winter months. The peak is mainly driven by 10 cases in January 2020 and 9 in January 2021, and it is possible that the association has arisen solely by chance. An alternative explanation is that the peak in January is an artefact of the industry's sampling strategy, e.g., that the sampling or reporting is intensified at the beginning of the year.

A limitation of our analysis is that it is correlational, and the causal bases for the associations are open to alternative interpretations. While we controlled for geographic and temporal covariates in the model, we cannot exclude that the association between delousing and *Pasteurella* was caused by some factors that were not included in the model. Hence, results should be considered hypotheses for further research, e.g., using molecular and experimental methods. Another limitation is that since pasteurellosis is not a notifiable disease, our data set probably does not include all diagnoses of *Pasteurella* in the studied period. Incomplete data coverage does not influence our conclusions about the associations between *Pasteurella* and delousing or other factors, but may have led to an underestimation of the odds for *Pasteurella*.

Implementing good practices during delousing operations, such as refreshing the water between batches in thermal treatments, has been suggested as a way to potentially reduce infection pressure by limiting pathogen buildup in the treatment environment (Nilsen, Gulla, and Colquhoun 2024). In addition, the industry has introduced autogenous vaccines against *Pasteurella* to mitigate outbreaks. In a 2023 survey on vaccine effectiveness, respondents who vaccinated their stocks against *Pasteurella* reported some success (Sommerset et al. 2024), although vaccine protection has not yet been fully documented.

## 5 Conclusions

The current study clearly indicates that while the presence of the bacterium itself is critical to the development of the disease, thermal and brushing/flushing based delousing methodologies appear to play a major role in the maintenance of the current pasteurellosis situation in western Norway. The results also indicate that freshwater‐based treatments may represent a favourable alternative to thermal and brushing/flushing based delousing treatments.

## Author Contributions

**Leif Christian Stige:** formal analysis, writing – original draft, writing – review and editing. **Duncan J. Colquhoun:** conceptualization, writing – original draft, writing – review and editing. **Victor H. S. Oliveira:** data curation, writing – original draft, writing – review and editing.

## Conflicts of Interest

The authors declare no conflicts of interest.

## Supporting information


Data S1.

## Data Availability

The diagnostics data analysed in this article is governed by agreements with data owners and not openly available.
