# Supplementary material for: Associations Between Delousing Practices and Pasteurellosis in Farmed Atlantic Salmon
Source: J Fish Dis. 2025 Jan 27;48(5):e14085. doi: 10.1111/jfd.14085 (PMC11976209; doi:10.1111/jfd.14085)
Supplement: Supplementary file 1 — Data S1. [file JFD-48-e14085-s001.docx]

Supplementary tables and figures

This supplemantery information file accompanies the article: “Associations between delousing practices and pasteurellosis in farmed Atlantic salmon” by Leif Christian Stige, Duncan J. Colquhoun and Victor H. S. Oliveira.

**Supplementary tables**

**Table S1. Text segments used to characterise non-medicinal delousing as thermal, mechanical or freshwater based on the free-text field in weekly reports from salmon farmers to the Norwegian Food Safety Authority.**

A. Thermal

- Optilicer (text segments: “tilic”, “ptlic”, “tlic”, “ptliz”, “ptil”, “ptiul”, “ptik”, “pili”, “potil”, “prtil”, “pti l”, “pitl”, “PTIL”, “ptril”, “ptiL”, “Ptil”, “ptlli”, “tlili”)

- Thermolicer (text segments: “ermo”, “hermo”, “ermi”, “ermli”, “ERMOLI”, “rmoli”, “herom”, “etmol”, “hernol”, “ermlo”, “ermel”, “herma”, “TL”, “hemolic”, “emolice”, “ermeolic”, “themro”, “thero”)

- Other descriptions of thermal delousing (text segments: “arm”, “ermisk”, “emperert”, “lunk”, “erms”, “THERM”, “hot water”, “term”, “erkolicer”, “hordalicer”)

B. Mechanical

- Hydrolicer (text segments: “drolicer”, “ydolicer”, “rolis”, “yrolicer”, “ydri”, “ydrol”, “yrdol”, “ro>Licer”, “ydro l”, “ro›Licer”, “ydroflow”, “Hydro”, “Hyrdo”, “hfc”)

- SkaMik (text segments: «kaMIk», «kami», «SKAMIK», «aMik», «kaMik», «Scamik»)

- Flatsesund (text segments: “FLS”, “fls”, “setsund”, “Fls”, “FSL”)

- Wellfighter (text segment: “ellfi”)

- Optiflush (text segment: “optifl”)

- Freshwell (text segment: “freshwell”)

- Other descriptions of mechanical delousing (text segments: “pyler”, “pyl”, “lush”, “SFI “)

C. Fresh water

- Various descriptions (text segments: «vann», «erskva», «ersvan», «vann i br», «ferskv», «ferska», «erks», «fv», «resh», «erkvan», «ANNBEH», «eskvan», «fw», «fersk vann»)

**Table S2. Summary of main model.**

Family: bernoulli

Links: mu = logit

Formula: Past ~ aar.factor + PO.factor + Past_before.factor + vlaks.factor2 + aTERM.factor + aMEK.factor + aFV.factor + (1 | lok.factor/pc.factor)

Data: Data.r (Number of observations: 13912)

Draws: 4 chains, each with iter = 4000; warmup = 2000; thin = 1;

total post-warmup draws = 8000

Group-Level Effects:

~lok.factor (Number of levels: 356)

Estimate Est.Error l-95% CI u-95% CI Rhat Bulk_ESS Tail_ESS

sd(Intercept) 0.42 0.29 0.02 1.07 1.00 863 1498

~lok.factor:pc.factor (Number of levels: 1161)

Estimate Est.Error l-95% CI u-95% CI Rhat Bulk_ESS Tail_ESS

sd(Intercept) 0.45 0.31 0.02 1.15 1.00 653 1166

Population-Level Effects:

Estimate Est.Error l-95% CI u-95% CI Rhat Bulk_ESS Tail_ESS

Intercept -9.02 0.71 -10.54 -7.75 1.00 1402 2296

aar.factor2019 0.53 0.49 -0.39 1.51 1.00 4950 4864

aar.factor2020 1.84 0.43 1.04 2.72 1.00 3397 4210

aar.factor2021 1.85 0.44 1.04 2.77 1.00 3228 4182

aar.factor2022 1.84 0.45 1.02 2.78 1.00 2759 3577

aar.factor2023 1.37 0.47 0.49 2.34 1.00 2992 4061

PO.factor3 0.47 0.31 -0.10 1.09 1.00 5544 5243

PO.factor4 0.48 0.32 -0.12 1.14 1.00 6505 5416

PO.factor5 -0.67 0.45 -1.58 0.20 1.00 8824 6629

Past_before.factor1 1.11 0.31 0.44 1.67 1.00 1465 2382

vlaks.factor22 0.37 0.55 -0.68 1.47 1.00 5577 4520

vlaks.factor23 1.93 0.45 1.09 2.90 1.00 4345 3891

vlaks.factor24 2.59 0.46 1.73 3.53 1.00 3857 3675

vlaks.factor25 3.10 0.46 2.28 4.08 1.00 2472 3080

aTERM.factorTRUE 0.93 0.18 0.58 1.27 1.00 12338 5793

aMEK.factorTRUE 0.55 0.26 0.04 1.06 1.00 14452 5751

aFV.factorTRUE -0.08 0.32 -0.75 0.51 1.00 15235 5639

Draws were sampled using sampling(NUTS). For each parameter, Bulk_ESS

and Tail_ESS are effective sample size measures, and Rhat is the potential

scale reduction factor on split chains (at convergence, Rhat = 1).

**Supplementary figures**


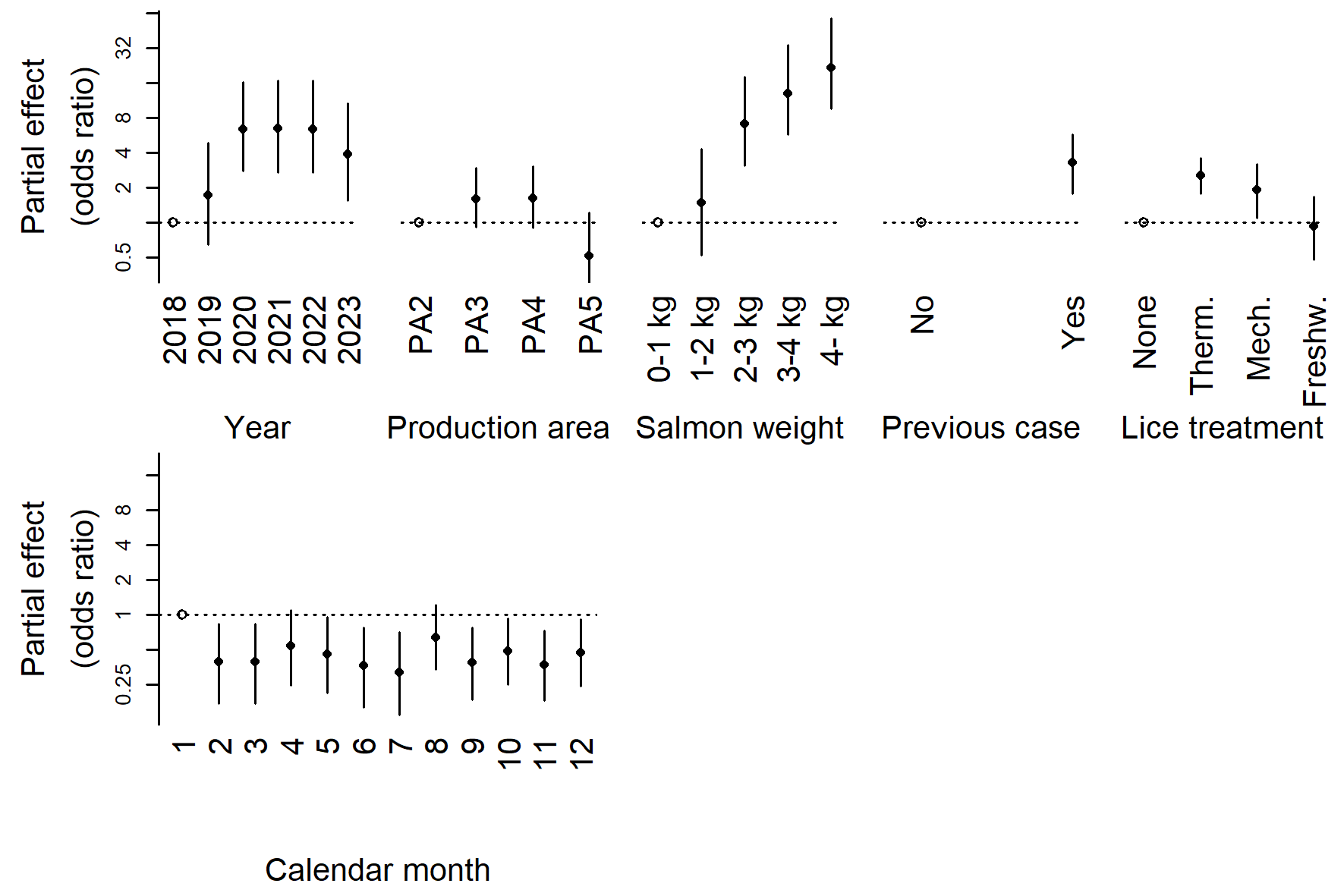


**Figure S1.**  Estimated covariate effects on the monthly probability of Pasteurella in a salmon farm in a model that includes calendar month as covariate. The covariates were otherwise as in the main model (**Fig. 3**). Effects are shown as odds ratios, which is the ratio for the probability of Pasteurella for a given covariate state compared to a reference state (indicated by a circle for each covariate). An odds ratio of 1 indicates no difference in probability of Pasteurella. Bands show 95 % credible intervals.

**
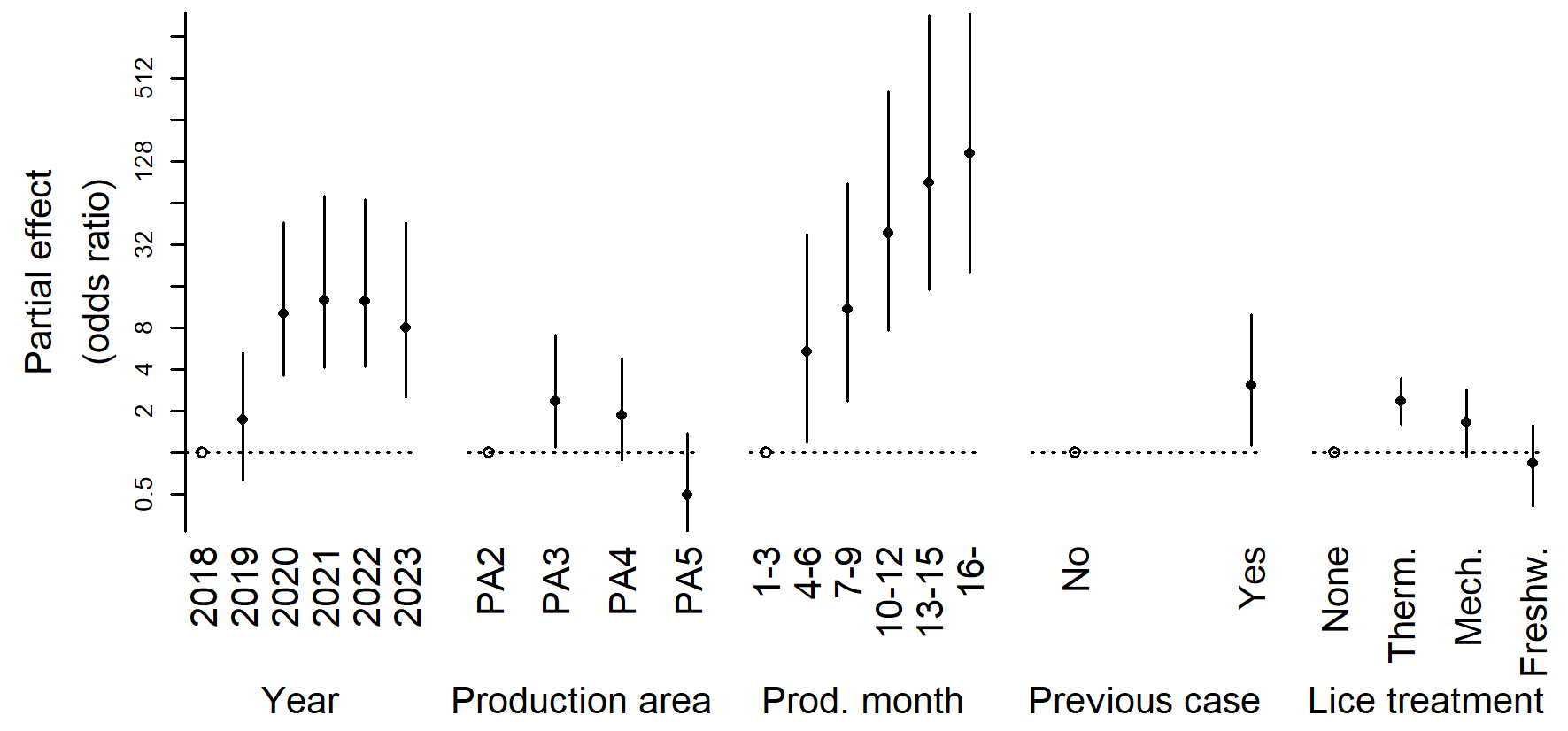
**

**Figure S2.**  Estimated covariate effects on the monthly probability of Pasteurella in a salmon farm in a model that includes production month instead of mean salmon weight as covariate. The covariates were otherwise as in the main model (**Fig. 3**). Effects are shown as odds ratios, which is the ratio for the probability of Pasteurella for a given covariate state compared to a reference state (indicated by a circle for each covariate). An odds ratio of 1 indicates no difference in probability of Pasteurella. Bands show 95 % credible intervals.

**
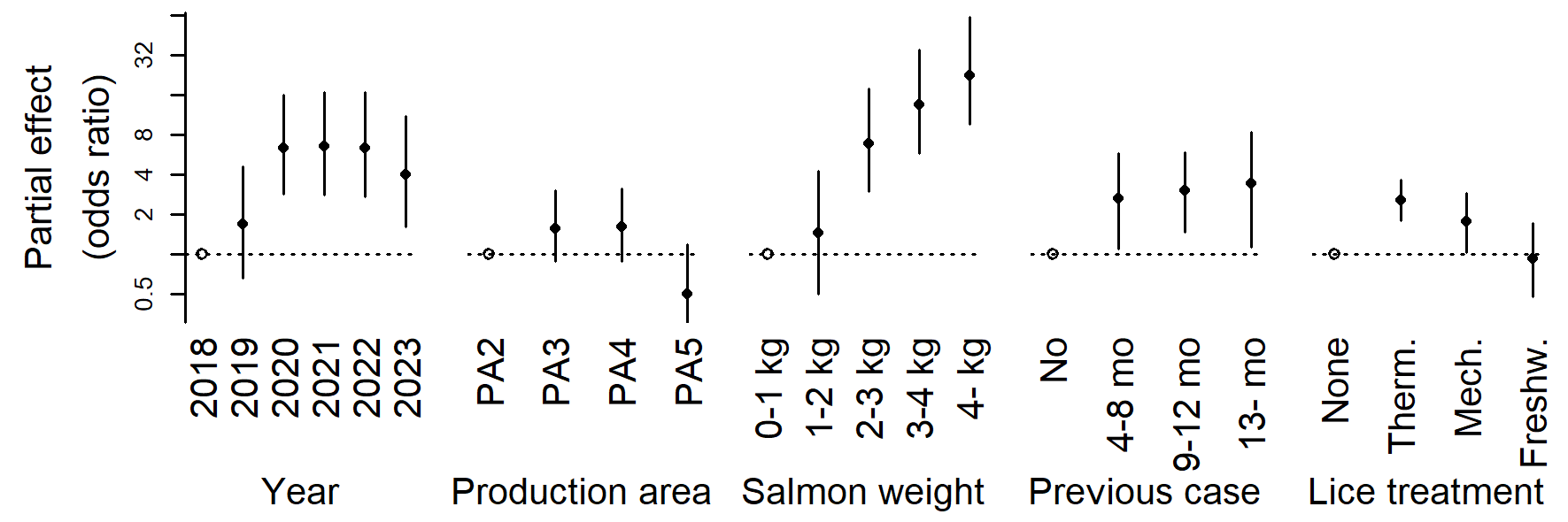
**

**Figure S3.**  Estimated covariate effects on the monthly probability of Pasteurella in a salmon farm in a model that includes fallowing time after a previous case as covariate (no Pasteurella in the previous production cycle at the farm; or Pasteurella and 4‒8, 9‒12 or 13+ months fallowing). The covariates were otherwise as in the main model (**Fig. 3**). Effects are shown as odds ratios, which is the ratio for the probability of Pasteurella for a given covariate state compared to a reference state (indicated by a circle for each covariate). An odds ratio of 1 indicates no difference in probability of Pasteurella. Bands show 95 % credible intervals.
